# Fabrication of near-invisible solar cell with monolayer WS_2_

**DOI:** 10.1038/s41598-022-15352-x

**Published:** 2022-07-04

**Authors:** Xing He, Yuta Iwamoto, Toshiro Kaneko, Toshiaki Kato

**Affiliations:** Graduate School of Engineering, Tohoku University, Sendai, 980-8579 Japan

**Keywords:** Two-dimensional materials, Materials for devices, Materials for energy and catalysis, Electrical and electronic engineering, Energy harvesting, Renewable energy

## Abstract

Herein, we developed a near-invisible solar cell through a precise control of the contact barrier between an indium tin oxide (ITO) electrode and a monolayer tungsten disulfide (WS_2_), grown by chemical vapor deposition (CVD). The contact barrier between WS_2_ and ITO was controlled by coating various thin metals on top of ITO (M_x_/ITO) and inserting a thin layer of WO_3_ between M_x_/ITO and the monolayer WS_2_, which resulted in a drastic increase in the Schottky barrier height (up to 220 meV); this could increase the efficiency of the charge carrier separation in our Schottky-type solar cell. The power conversion efficiency (PCE) of the solar cell with the optimized electrode (WO_3_/M_x_/ITO) was more than 1000 times that of a device using a normal ITO electrode. Large-scale fabrication of the solar cell was also investigated, which revealed that a simple size expansion with large WS_2_ crystals and parallel long electrodes could not improve the total power (P_T_) obtained from the complete device even with an increase in the device area; this can be explained by the percolation theory. This problem was addressed by reducing the aspect ratio (width/channel length) of the unit device structure to a value lower than a critical threshold. By repeating the experiments on this optimized unit device with an appropriate number of series and parallel connections, P_T_ could be increased up to 420 pW from a 1-cm^2^ solar cell with a very high value (79%) of average visible transmission (AVT).

## Introduction

Transparent solar cells (TSCs) have attracted considerable attention as they can overcome the limitations of traditional non-transparent solar cells^1^, which can convert diverse components, such as architectural windows, agricultural sheds, glass panels of smart devices, and even human skin into energy harvesting devices. Despite the recent developments in TSCs with perovskite^2^ and organic semiconductors^3–5^, the average visible transparency (AVT) of these resources is lower than 70%; thus, TSCs with very high AVT values (> 70%), here we call ‘‘near invisible solar cells (NISCs)’’, is still challenging. To the best of our knowledge, NISCs have been realized only by combining several devices. Transparent luminescent solar concentrators (TLSCs)^6,7^ absorb UV or IR and generate bright luminescence, which propagates to the edge of the TLSC by internal reflection. Electrical power generation can be achieved at the edge of the TLSCs using a conventional non-transparent Si solar cell, resulting in a very high AVT (> 80%). However, a non-transparent part is always required at the edge of the device, suggesting that scalability issues could arise^6,7^. Recently, TSCs with relatively high AVT values have been reported in AZO-embedded ZnO/NiO/AgNW^8^ (70%), dye^9^ (75%) and ClAlPc:C_60_^10^ (77.45%). These can be candidates of NISCs.

Two-dimensional (2D) transition metal dichalcogenides (TMDs), especially for monolayer- and few layered TMDs, including suitable band gaps in the visible light range and highest absorption co-efficiency per thickness^11,12^, are considered to be one of the most promising materials for the fabrication of NISCs. To date, many TMD-based solar cells have been investigated by employing pn junctions^13–17^, heterojunctions^18–20^, Schottky junctions^21^ etc. (Tables S1 and S2). However, most of them are non-transparent owing to the use of opaque Si substrates and non-transparent metal electrodes^13–17,19^. Even though devices of μm-scale can attain a certain power conversion efficiency (PCE), the total power (P_T_) obtained from the whole device within the same substrate is too low to be applied^22,23^ in devices because of their lack of scalability. In our previous study, a highest PCE of 0.7% was achieved using a triple-layer TMD^21^ with a Schottky junction structure. This Schottky junction structure can be expanded and applied even on a polyethylene naphthalate (PEN) substrate at cm-scales. However, there was a trade-off between transparency and P_T_ because non-transparent metal electrodes were utilized. Thus, transparent electrodes and monolayer TMDs should be employed to realize TMD-based NISCs with a relatively high P_T_.

In this study, we successfully fabricated an NISC using ITO and monolayer tungsten disulfide (WS_2_) as transparent electrodes, and photoactive layer, respectively. The contact issue between ITO and WS_2_ was investigated, revealing that a pure ITO–WS_2_ junction possessed a relatively low Schottky barrier height (*Φ*_B_) (< 10 meV), and a thin Cu-coated ITO electrode with an insertion of WO_3_ (WO_3_/Cu/ITO) could increase *Φ*_B_ up to approximately 220 meV in contact with WS_2_; this can be useful for carrier collection and generation within a Schottky-type solar cell, respectively. Furthermore, it was found that an appropriate architectural design would be vital to scale up the TMD-based solar cell to avoid an unexpected drop in the open circuit voltage (V_OC_), which can be overcome by controlling the aspect ratio of the unit device. It was demonstrated that P_T_ could reach up to 420 pW from a 1-cm^2^ device with a high AVT of 79%; thus, the fabrication of NISC was first realized by using TMD.

## Results and discussion

### Work function control of ITO

**Figure 1:**
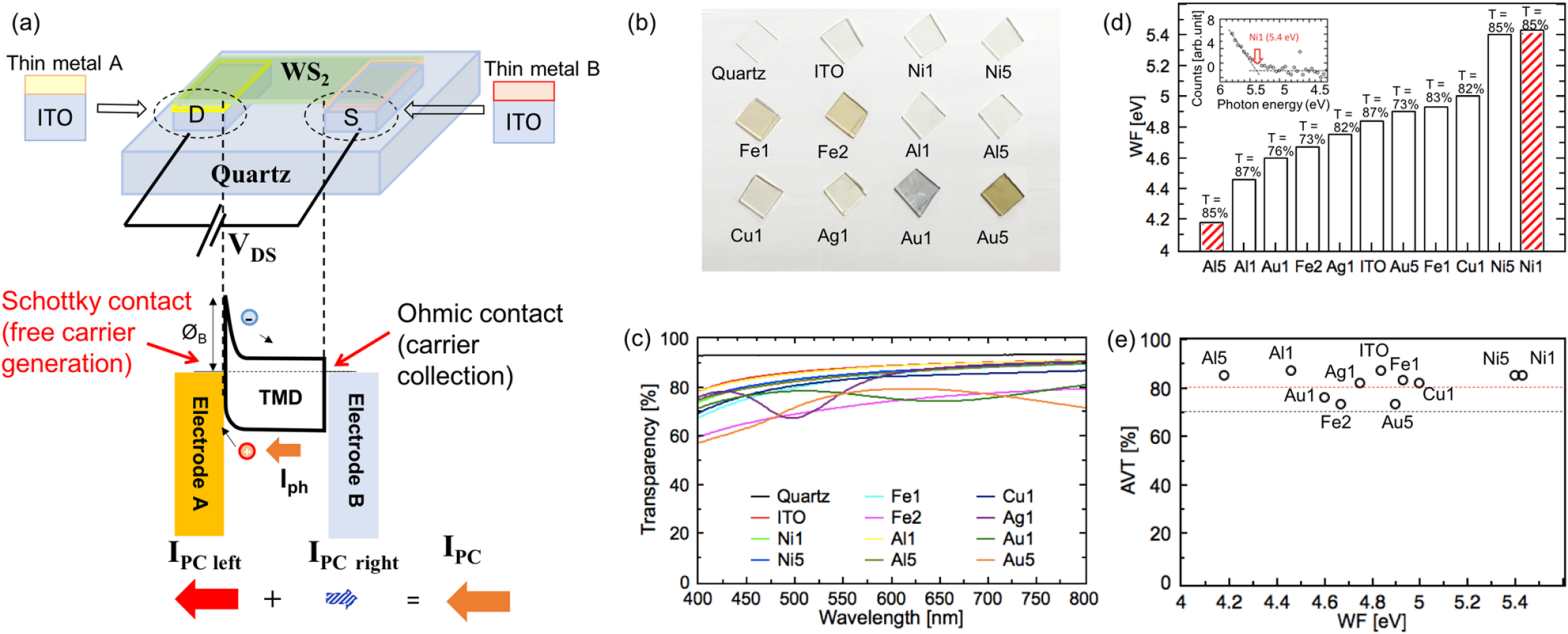
(**a**) Schematic illustration of device structure and ideal optimal band structure for the transparent Schottky solar cell. (**b**) Images of samples for WF and AVT measurement. ITO was sputtered on quartz substrate and a thin metal film was coated on the top of ITO. (**c**) Transparency spectra of quartz, ITO/quartz, and Mx/ITO on quartz (Mx = Ni1, Ni5, Fe1, Fe2, Al1, Al5, Cu1, Ag1, Au1, and Au5). (**d**) Plot of WF for Mx/ITO measured by PYS. Inset in (**d**) shows typical photoemission features of Ni1/ITO and fitting curve to obtain the WF. (**e**) Scatters of transparency and work function of tested Mx/ITO.

The device structure and ideal band structure of the Schottky-type solar cell are shown in Fig. 1a. The discrepancy in the work function (WF) between electrode A and the semiconductor produces a built-in potential, which separates the photogenerated electron–hole pairs. Once the generated carriers travel to the opposite electrode, power generation can be realized. In our previous study, it was proven that the greater the WF difference between the asymmetric electrodes, the higher the resultant PCE; this is consistent with this power generation model. Thus, in this study, the WF control of ITO was the first objective towards obtaining an optimal band structure for the Schottky-type solar cell. Regarding to the interface, it should be notice that, it is better to avoid other interface impurity, because sometimes it could affect the interface charge and energy distribution that lead to some undesired surface recombination, reducing the separation efficiency of carriers at the interface barrier, thereby reducing the V_OC_.

To modulate the WF, different types of thin metal films were coated on ITO. After an Mx coating (Fig. 1b, c; M is a metal and x is thickness in nm), some Mx/ITOs can maintain a high AVT of greater than 80%, such as Ni1, Ni5, Fe1, Al1, Al5, Cu1, and Ag1; this indicates their potential to be used as electrodes in TSCs. The WF of the Mx/ITO was then measured using photoelectron yield spectroscopy (PYS). It was found that the WF of the Mx/ITO varied in the range of 4.2– 5.4 eV (Fig. 1d). Moreover, most of them had a transparency of more than 80%, indicating that the WF of ITO was successfully modulated by maintaining the original high transparency of ITO (Fig. 1e). As a monolayer WS_2_ grown by CVD is naturally n-doped by impurities and the WF is measured to be around 4.9 eV, Mx/ITOs with WF higher and lower than 4.9 eV are promising candidates as transparent electrode with Schottky contacts (Ni1, Ni5, Cu1 and Fe1), and Ohmic-like contacts (Al5, Al1, Ag1, ITO), respectively against a monolayer WS_2_.

### Schottky barrier height measurement and control

**Figure 2:**
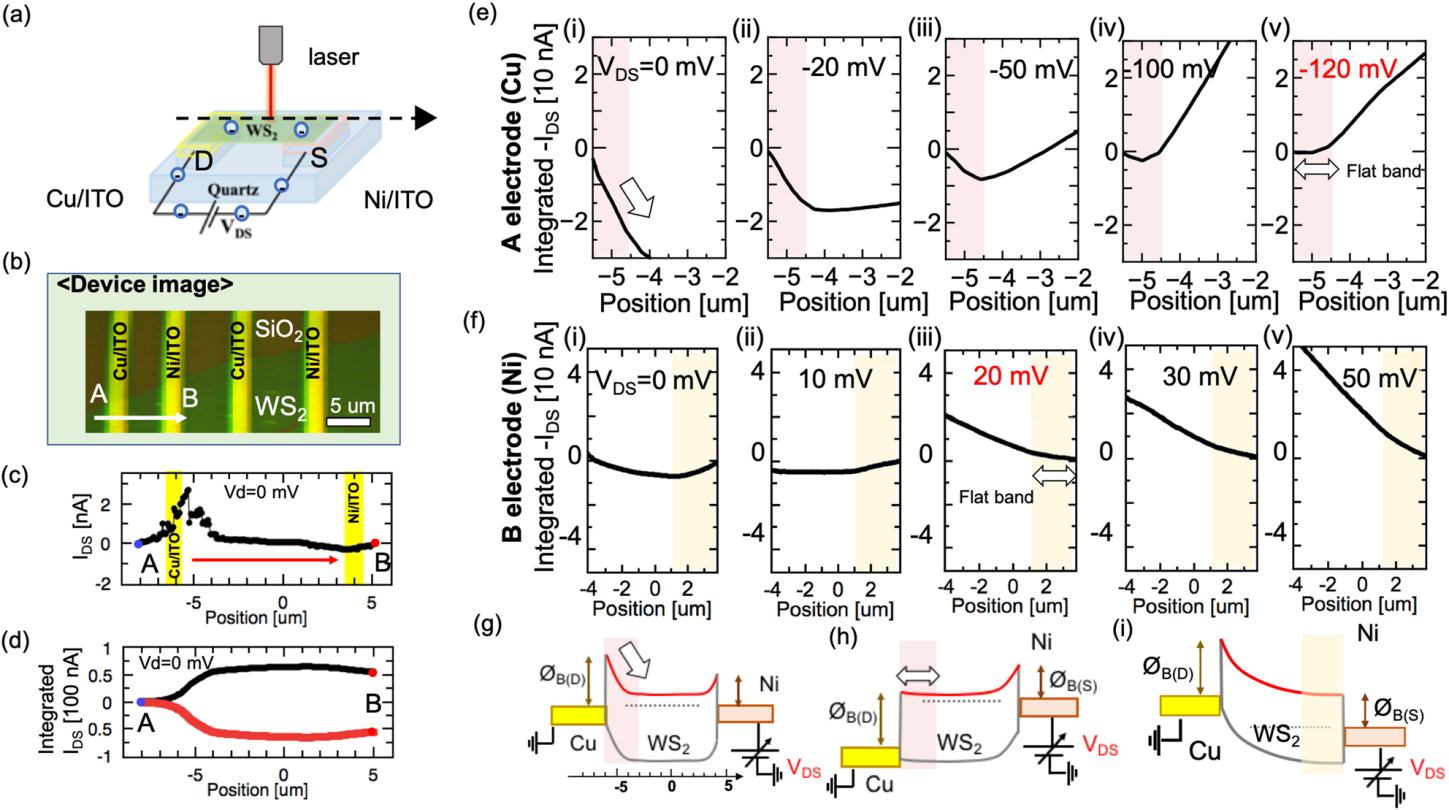
(**a**) Schematic illustration of spatially resolved photoexcited charge-carrier mapping for the device with asymmetric electrodes: Cu/ITO and Ni/ITO. (**b**) Images of the device under a microscope. (**c**) Photocurrent line scan of the device. (**d**) Integrated photocurrent based on photocurrent in (**c**) as the black line; the red line comes from inverse treatment of the y-axis, which represents the potential profile in the conduction band as shown in (**g**). (**e**), (**f**) (i–v) Profile of integrated I_DS_ at (**e**) Cu/ITO and (f) Ni/ITO side under different values of V_DS_, respectively. (**g**)–(**i**) Typical band structure of (**g**) original and flat band in (**h**) Cu and (**i**) Ni side.

The purpose of controlling the WF was to control *Φ*_B_ in both the contacts. Thus, it is important to understand the band structure of a real device. Here, the spatially resolved photoexcited charge-carrier mapping (SPCM) method was used to measure the potential profile of the real device^24^. By using this method, the detailed band structure between the Mx/ITO and TMD could be obtained under the ambient condition, which is the operating condition of the solar cell. The monolayer WS_2_ was used as the photoactive channel material in contact with Cu/ITO (left) and Ni/ITO (right) (Fig. 2a and b). The methodology to obtain the potential profile is outlined below: (1) A 633 nm cw excitation light was delivered by a 100 objective (laser spot size ~ 1um) and focused on the sample to be scanned over the samples (electrode width ~ 2um, channel length > 3um) in a form of line/fixed position. A line scan from the drain to source electrode, that is, from Point A to Point B was taken, measuring all the spots within this scan. Synchronous detection of photocurrent (the source-drain current (I_DS_)) was achieved (Fig. 2c); (2) I_DS_ was integrated from Point A to B (Drain to Source) (Fig. 2d, black line); (3) The y-axis was switched to present the electron negative potential (potential A is always zero) (Fig. 2d, red line), which could be considered to be the same as that of the conduction band (Fig. 2g) because of the simple approximation between the potential and carrier generation^24^.

**Figure 3:**
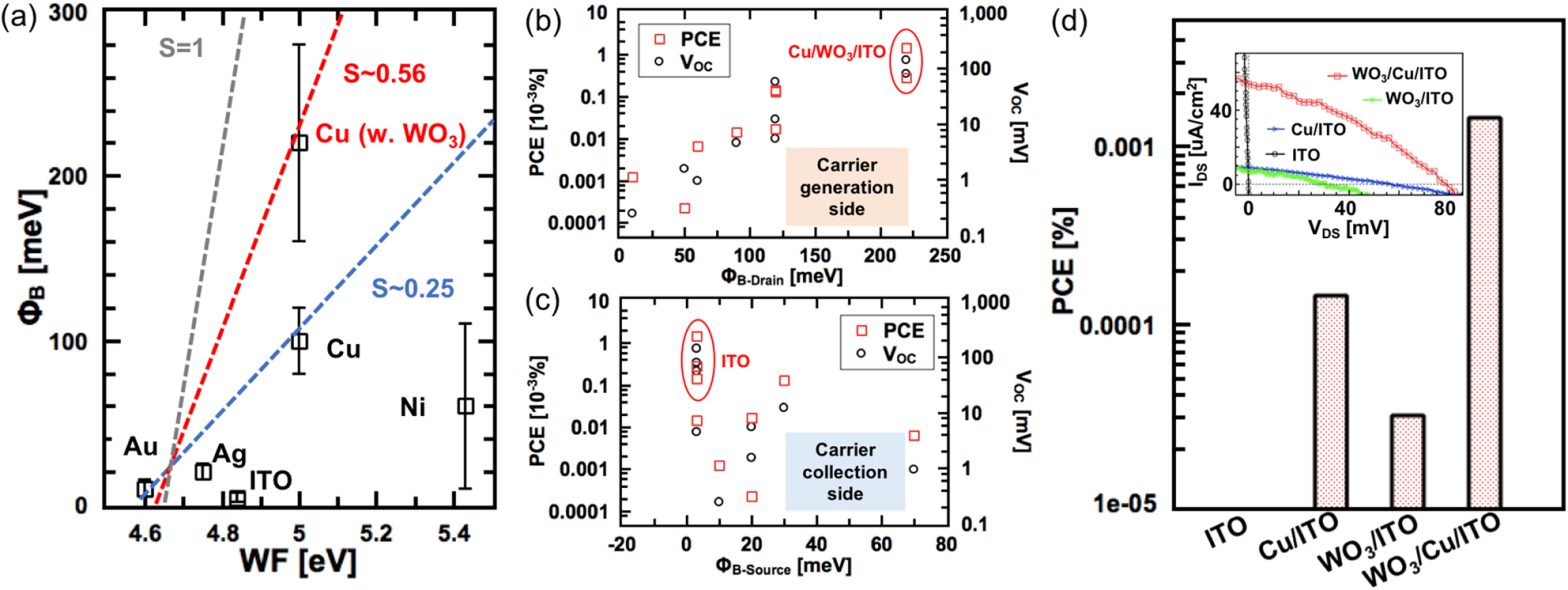
(**a**) Experimentally determined Schottky barrier height vs WF for different metal coated ITO with or without WO_3_. Dashed lines represent theoretical calculations with different value of S (gray: S = 1, red: S = 0.56, blue: S = 0.25). (**b**), (**c**) Power conversion efficiency and open circuit voltage vs Schottky barrier height in the contact of (**b**) drain side and (**c**) source side. (**d**) PCE of different transparent Schottky solar cells fabricated by different kinds of symmetric or asymmetric electrodes. Insert in (**d**) is the typical I_DS_-V_DS_ curve of these solar cells.

First, we focused on the Cu/ITO side under different source-drain bias voltages (V_DS_) (Fig. 2e (i)–(v)). The negative slope of the potential near the Cu/ITO interface decreased with increasing V_DS_ and became almost flat at 120 mV, indicating that *Φ*_B_ at the Cu/ITO side was approximately 120 meV (Fig. 2h). Then, the slope changed from positive to negative slope at approximately 20 mV at the Ni/ITO side (Fig. 2f (i)–(v)), indicating that *Φ*_B_ on the Ni/ITO side was approximately 20 meV (Fig. 2i). Using the SPCM method, different types of Mx/ITO devices were systematically measured, and each *Φ*_B_ was carefully estimated from the V_DS_ dependency of the SPCM (Table S3). The correlation between *Φ*_B_ and the WF for different Mx/ITO interfaces is displayed in Fig. 3a. Several sets of devices tested and error bars are added (Fig. S1). It was found that all the data points except those for Ni followed a linear tendency in that *Φ*_B_ was proportional to the WF following the equation $\Phi_{B}$ = S_F_ × $(\Phi_{{\text{m}}} - {\upchi })$*,* where S_F_, $\Phi_{{{\text{m}},}}$ and ${\chi }$ are Fermi level pinning (FLP) factor, the WF of the conductive material and electron affinity of the semiconductor, respectively. Through curve-fitting our data using this formula, S_F_ was calculated to be approximately 0.25 (blue dashed line in Fig. 3a), which was consistent with the results for non-transparent bulk metal contact against TMDs obtained by other groups (S_F_ = 0.1–0.3)^25,26^. According to this equation, *Φ*_B_ of Ni should be approximately 200 meV, which is completely different from the experimental results (*Φ*_B_ = 10–110 meV). It is known that metals with stronger/weaker binding energies (E_b_) owing to their shorter/longer bonding distances (d) are considered to cause a stronger/weaker FLP effect^27^. Based on the previous theoretical calculation of the binding energy against TMDs, the E_b_ of Ni was obtained to be 510 meV, which was much higher than that of Cu (400 meV), Ag (350 meV), and Au (300 meV)^27^, thus denoting a stronger FLP effect of Ni as compared to Cu, Ag, and Au. This relatively stronger FLP effect may account for the much lower *Φ*_B_ than the theoretical value for Ni.

Based on the above, it can be hypothesized that a slight increase/decrease in d (E_b_) could weaken the FLP effect and increase *Φ*_B_, resulting in an improved performance of the Schottky-type solar cell. To confirm this hypothesis, a thin WO_3_ (a few nm) layer was inserted as an insulating oxide layer. WO_3_ is a promising insulating material with high work function, good hole mobility and high transparency^28–30^ were used as a interlayer between metal and semiconductor, to improve contact^31^ and device performance, such as work as a buffer layer in photovoltaic devices^10,28^ and improve the performance of transparent organic light emitting diode^32^. The WO_3_ layer can be deposited by thermal evaporation, which is compatible to thermal evaporation of anode Au or Ag.

As a result, *Φ*_B_ of WO_3_/Cu/ITO increased up to 220 meV (Fig. S2), which was much higher than that of Cu/ITO (*Φ*_B_ = 120 meV) (Fig. 3a); this confirms the significant effect of WO_3_ insertion on *Φ*_B_. The S_F_ of WO_3_/Cu/ITO was calculated to be approximately 0.56, which indicates a weakening of the FLP effect. There is also a possibility that WO_3_ provides a carrier filtering effect and thus affects the performance of solar cells. Other oxide films and film thicknesses will be tried and clarified in the future.

Because it is now possible to control *Φ*_B_ by tuning the surface of the ITO electrode, the PCE of our solar cell devices could be measured. The relationship between *Φ*_B_ and PCE is summarized in Fig. 3b, c. The PCE and V_OC_ increased with *Φ*_B_ at the drain side (*Φ*_B-drain_) (Fig. 3b), and decreased with *Φ*_B_ at the source side (*Φ*_B-source_) (Fig. 3c); this suggested that the higher PCE and V_OC_ originated from the higher *Φ*_B-drain_ and lower *Φ*_B-source._ Because the drain and source electrodes were designed for carrier generation and collection electrode of the Schottky-type solar cell, these results agreed well with those of our power generation model. Among these solar cells, WO_3_/Cu/ITO with the highest *Φ*_B-drain_ exhibited the best PCE (1.45 $\times$ 10^–3^%), which was 10 times higher than that of Cu/ITO (1.44 $\times$ 10^–4^%), and more than 1000 times that of pure ITO (Fig. 3d) (Fig. S3).

### Scale-up of highly transparent Schottky solar cells

Even though a very high PCE could be obtained from a small device at a µm-scale, the P_T_ of the entire device would be considerably limited by the device size. Thus, P_T_ is an important parameter for determining the potential of a solar cell for practical applications. As the solar cell study using TMDs is in a relatively nascent phase, P_T_ has not been discussed till date. In this study, we attempted to increase P_T_ to a practical level (more than 100 pW)^23^ by scaling up the device. However, it can be seen that scaling up by increasing the channel width and number of parallel connections cannot effectively increase P_T_, and may sometimes cause the P_T_ to drop instead (Fig. S4), indicating that it is necessary to choose a suitable architectural design to scale up the TMD-based solar cells. Here, some concrete strategies adopted for the scale-up include (1) designing the structure of the unit device (UD); (2) exploring parallel connections (named as unit module A (UDM-A)); (3) investigating series connections (named as unit device module B (UDM-B)); and (4) combining parallel and series connections (named as unit device module C (UDM-C)). The optimization of the architecture of each device is vital.

**Figure 4:**
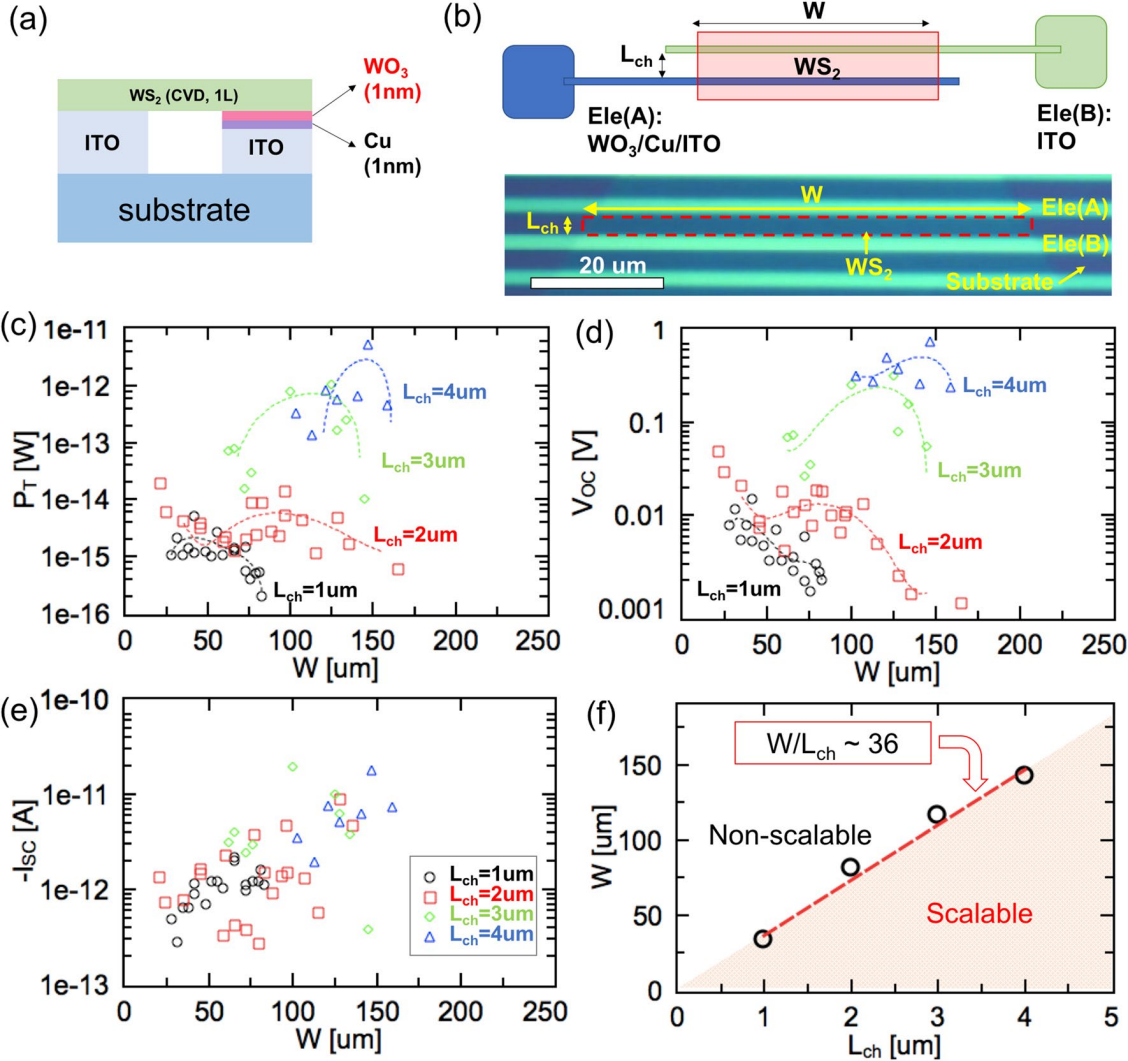
(**a**) Structure of devices used for architecture design of the UD. (**b**) Schematic of UD structure. (**c**)–(**e**) P_T_, V_OC_, I_SC_ of unit devices with different L_ch_ and W. (**f**) Critical width for device with different L_ch_.

As a first step in designing a unit device (UD) structure, solar cells with various widths (W) and channel lengths (L_ch_) were fabricated (Fig. 4a, b). The performance of the Schottky-type solar cells has been mainly discussed in terms of three aspects: P_T_, V_OC_, and short circuit current (I_SC_). When L_ch_ = 1 μm, P_T_ increased with W up to 33.5 μm (Fig. 4c). However, when W was larger than 33.5 μm, P_T_ significantly decreased with W, that is, there was a threshold value of the critical width (W_th_) essential to maintain a high P_T_. The drop in P_T_ was mainly owing to V_OC_ dropping (Fig. 4d, e). Coincidentally, a similar tendency was observed in the devices with L_ch_ = 2, 3, and 4 μm, with different W_th_ values of 81.3, 116.3 and 142.0 μm, respectively. Interestingly, there was an approximately linear relationship between W_th_ and L_ch_ (Fig. 4f), indicating that the aspect ratio of the device (W/L_ch_) was critical to designing large-scale solar cells with TMDs, and it should be lower than approximately 36. This could be explained by the decrease in the parallel resistance. The UD can be treated as a combination of several small channels connected in parallel, and each channel has a shunt resistance of R_sh_(i) (i = 1,2.., *n*) (Fig. S5). The total shunt resistance (R_sh-total_) would depend on the resistance of each channel (1/R_sh-total_ = $\Sigma$(1/R_sh_ (i)). If channel (i) contains a metal-like pass, R_sh_(i) would be very low, resulting in a low R_sh-total_ and a low V_OC_. Candidates that would cause a low R_sh_(i) are possibly impurities, such as the 1 T phase of TMD or other impurities existing in TMD, which are inevitably induced by chemical vapor deposition or mechanical exfoliation^33^. The presence of impurities leads to the formation of band tail localized states, thereafter, percolation transport would arise in 2D disordered materials with energy variations along the current-carrying path^34,35^. When W increased, the possibility that the unexpected metal-like pass involved within the photoactive channel would increase, resulting in a low R_sh-total_; this is known as the percolation model^36,37^, and can explain the existence of W_th_ and the linear correlation between W_th_ and L_ch_. Hereafter, we use L_ch_ = 2 μm and W = 10 μm to avoid unexpected V_OC_ drops to scale up the TMD-based Schottky-type solar cell.

To increase P_T_, we attempted to connect multiple UDs in parallel. With the increasing number of parallel connections (N_pa_) of UD, P_T_ increased up to N_pa_ = 11 (Fig. S6). However, a further increase in N_pa_ caused a sharp drop in V_OC_, resulting in a lower P_T_; this could also be explained by the aforementioned percolation model.

Once we found a suitable structure for UD and N_pa_, the effect of the number of series connections (N_se_) was investigated. When N_se_ was less than 4, P_T_ and V_OC_ increased (Fig. S7). When N_se_ was larger than 4, the P_T_ drop mainly came from the decrease in not only V_OC_ but also I_SC_, implying an excessive carrier loss owing to the longer travelling distance of the carriers. It may happen the recombination of carrier when carrier density increase rather than the critical threshold. At current stage, we have not been able to conduct the recombination lifetime measurements, which will keep as one of the future works.

**Figure 5:**
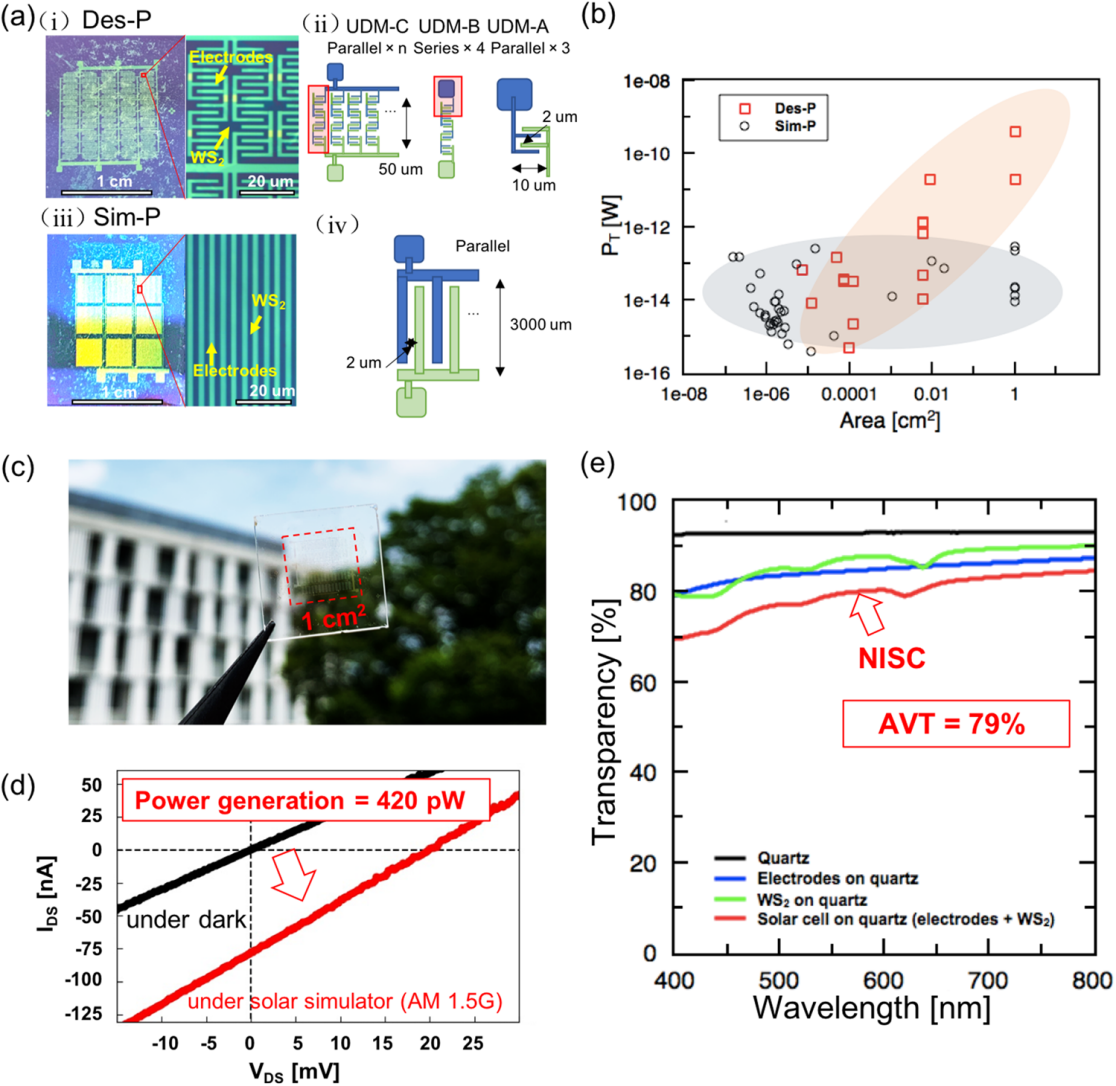
(**a**) (i) Image for solar cell with designed pattern (Des-P), (ii) structure diagram of designed pattern, (iii) image for solar cell with simple pattern (Sim-P), and (iv) structure diagram of simple pattern. (**b**) Plot of P_T_ for solar cells with different areas in Sim-P or Des-P. (**c**) Optical image of a highly transparent solar cell (**d**) I_DS_-V_DS_ curve of highly transparent solar cell under dark or solar simulator. (**e**) Transmission of the NISC with comparison of WS_2_ on quartz, electrodes on quartz, and pure quartz.

The combination of parallel and series connections, as well as further paralleling of UDM-B (unit device module C (UDM-C)) was also investigated. It was found that P_T_ increased by more than 10^6^ times than that of UD upon repeatedly connecting 18,750 units of UDM-B at the cm-scale (Fig. 5a (i–ii) and 5b). As a control experiment, simply scaled up devices (L_ch_ = 2 μm and W = 3000 μm; all parallel connections) were fabricated, in which P_T_ did not increase even with a device area 10^6^ times larger than that of UD (Fig. 5a (iii–iv) and b) (Fig. S8). These results indicate that the appropriate series–parallel composite design is of significant importance to P_T_ optimization of TMD-based NISCs.

Finally, a similarly optimized device designed on an SiO_2_/Si substrate ((WO_3_/Cu/ITO-ITO) and architecture (UDM-C: UD × 3 parallel connections (UDM-A) × 4 series connections (UDM-B) × 18,750 parallel connections)) were fabricated on a quartz substrate (Fig. 5c) (Fig. S9). As a result, the AVT and P_T_ reached up to 79% and 420 pW, respectively (Fig. 5d and e), which can drive several real devices^23^. To the best of our knowledge, this is the first investigation on realizing an NISC with TMD, and with the P_T_ obtained being the highest value for a solar cell using a monolayer or a few layers of TMD regardless of the AVT (Table S2).

## Conclusion

A monolayer WS_2_ based NISC was successfully fabricated. The work function of the transparent ITO electrodes was modulated by a thin metal film coating without sacrificing the high transparency of ITO. A systematic investigation of *Φ*_B_ measurement with SPCM revealed that *Φ*_B_ was successfully controlled by the WF control of ITO and the S_F_. A high *Φ*_B_ (~ 120 meV) was obtained using the Cu/ITO electrode. *Φ*_B_ could be further increased up to ~ 220 meV by inserting a thin film of WO_3_ between the TMD and Cu/ITO electrode, and this can be explained by the reduced FLP effect. The PCE of the device with the highest *Φ*_B_ (WO_3_/Cu/ITO) was more than 1000 times higher than that of the bare ITO electrodes. A suitable architectural design for large-scale device fabrication was also investigated. It was found that the aspect ratio (W/L_ch_) of the TMD device should be lower than the critical value of approximately 36, which can be determined by the percolation model. By further scaling up the device size by considering an optimal series–parallel connection structure, an extremely high transparency of 79% could be realized, with P_T_ reaching up to 420 pW; this is the highest value within a TMD based solar cell with a few layers. These findings can contribute to the study of TMD-based NISCs from fundamentals to truly industrialized stages.

## Experiment and method

### Structural characterization of the TMD

The structures of the TMD were characterized by optical microscopy, Raman spectroscopy, and PL spectroscopy (LabRam HR800, HORIBA JOBIN YVON, Japan), and spatial mapping measurements with a 532-nm laser (Fig. S10).

### Device fabrication on SiO_2_/Si or quartz substrates

WS_2_ was synthesized by thermal CVD using WO_3_ as the tungsten source^38,39^. Ar was used as the carrier gas at a flow rate of 150–500 sccm. Sulfur (15 mg) was placed in a CVD oven, and WO_3_ (9 mg) on a quartz boat was set 15 cm downstream from the center of the CVD furnace. NaCl (1.5 mg) was mixed with WO_3_ to enhance evaporation. Then, conventional electron beam lithography, microwave-plasma sputtering of ITO (Fig. S11), vacuum evaporation of metal or metal oxide, and lift-off were used to fabricate the symmetric and asymmetric devices. For the small-scale and large-scale suspended device fabrication, the CVD-grown WS_2_ was covered with a water-soluble polymer and transferred to the asymmetric electrodes by a homemade transfer system with a micro positioner and microscope. The polymer was carefully removed by soaking it in water to obtain a suspended WS_2_ device between the asymmetric electrodes on an SiO_2_/Si substrate or a large transparent quartz substrate.

### Work function analysis

The work functions of Mx/ITO were measured by photoelectron yield spectroscopy (M20 KV-200, BUNKO-KEIKI, Japan)^40^. ITO (~ 40 nm) was sputtered on a quartz substrate; thereafter, thin metal films (1–5 nm) were prepared by thermal evaporation, which was the same as that used for device fabrication.

### AVT measurement

AVT was measured using UV–Vis-NIR spectroscopy (V-7200HK, JASCO, Japan) for vis-transparency measurements. AVT was calculated as $AVT = \frac{\smallint T\left( \lambda \right)P\left( \lambda \right)S\left( \lambda \right)d\lambda }{{\smallint P\left( \lambda \right)S\left( \lambda \right)d\lambda }}$, where λ is the wavelength; T is the transmission; P is the photopic response; and S is the solar photon flux (AM1.5G) for window applications, or 1 for other applications^1^.

### Solar cell performance measurements

The I_DS_–V_DS_ measurements were performed using a semiconductor parameter analyzer (HP 4155 C, Agilent, Japan) and a solar simulator (HAL-320, 300 W Xenon ramp with an AM1.5 filter, Asahi Spectra, Japan). The I_DS_–V_DS_ curves for the estimation of accurate PCE were measured with a reverse-to-forward bias (− 1 to 1 V_DS_), 1-mV step size, and 20-ms dwell time; however, no difference in I_DS_–V_DS_ curves was observed between the forward and reverse sweep directions. The power of the solar simulator (1000 W/m^2^) was calibrated using an AIST-certified standard solar cell (AK-100, KONICA MINOLTA Inc., JAPAN). We used the total area of the suspended WS_2_ between the two electrodes as the active area for the estimation of PCE in a small-scale device (~ μm^2^).

### Schottky barrier height measurements

The Schottky barrier height was measured using a homemade spatially resolved photoexcited charge-carrier mapping (SPCM) system. Line profile and mapping measurements of the photocurrent were performed using a homemade current probe system combined with a Raman and PL mapping measurement system (SPEX HR 640, HORIBA JOBIN YVON, Japan). The laser wavelength was 633 nm, and the spot size was ∼1 μm (× 100 objective).

## Supplementary Information

Supplementary Information.
