# Supplementary material for: Fabrication of near-invisible solar cell with monolayer WS2
Source: Sci Rep. 2022 Jul 4;12:11315. doi: 10.1038/s41598-022-15352-x (PMC9253307; doi:10.1038/s41598-022-15352-x)
Supplement: Supplementary file 1 — Supplementary Information. [file 41598_2022_15352_MOESM1_ESM.docx]

*Supporting information of*

**Fabrication of near-invisible solar cell with monolayer WS_2_**

Xing He^1^, Yuta Iwamoto^1^, Toshiro Kaneko^1^, and Toshiaki Kato^1,*^

*^1^Graduate School of Engineering, Tohoku University, Sendai 980-8579, Japan*

**Corresponding Author. e-mail: kato12@tohoku.ac.jp*

**Table S1|** Types and basic features of solar cells with thick TMD.

| **Type** | **Material** | **Layer**  **number** | **PCE** | **Device**  **size** | **Optical transparency** | **Ref.** |
| --- | --- | --- | --- | --- | --- | --- |
| PN  (Dual gate) | MoSe_2_ | 10L | 14%  (solar simulator) | μm-scale | X  (Si substrate) | S1 |
| PN  (Plasma doping) | MoS_2_ | ~120 nm | 2.8%  (Solar simulator) | μm-scale | X  (Au substrate) | S2 |
| Hetero  (Lateral) | MoS_2_/  WSe_2_ | 7.06/4.51 nm  (10L/6L) | ~10%  (19.1 W/m^2^) | μm-scale | O  (glass substrate) | S3 |
| Hetero  (Lateral) | n-MoS_2_/  p-InP | 6L (MoS_2_)/- | 0.11%  (solar simulator) | mm-scale | X  (Si substrate) | S4 |
| PN  (MoO_x_ Doping) | WS_2_ | < 90 nm | 1.55%  (solar simulator) | μm-scale | X  (Si substrate) | S5 |
| Schottky | MoS_2_ | 50 nm | 2.5%  (532 nm, 1000W/m^2^) | μm-scale | X  (Si substrate) | S6 |
| Schottky | WSe_2_ | 25 ~ 185 nm | 1.7%  (solar simulator) | μm-scale | X  (Si substrate) | S7 |

**Table S2|** Types and basic features of solar cells with monolayer and TMD with a few layers.

| **Type** | **Material** | **Layer**  **number** | **Total power** | **Device**  **size** | **Optical**  **transparency** | **Ref.** |
| --- | --- | --- | --- | --- | --- | --- |
| PN  (Dual gate) | WSe_2_ | 1L | 170 pW  (Laser, 640 nm,  48000 W/m^2^) | μm-scale | X  (Si substrate) | S8 |
| PN  (Dual gate) | WSe_2_ | 1L | 9 pW  (halogen lamp,  1400 W/m^2^) | μm-scale | X  (Si substrate) | S9 |
| Liquid  junction  (Vertical) | MoS_2_,  WSe_2_ | 1L (MoS_2_)  1L (MoS_2_)/  1L (WSe_2_)  1L (WSe_2_) | –  (532 nm,  20.8 W/m^2^) | μm-scale | X  (ITO substrate, Pt electrode) | S10 |
| Schottky  (lateral) | WSe_2_,  WS_2_ | 2L, 3L | 0.023 pW  (solar simulator,  1000 W/m^2^) | cm-scale | O  (PEN substrate,  Sparse scattered pattern) | S11 |
| Schottky | WS_2_ | 1L | 420 pW  (solar simulator,  1000 W/m^2^) | cm-scale | O  (Quartz substrate，  Dense integrated pattern) | This  work |

**Table S3|** Schottky barrier height of Mx/ITO, as measured by the SPCM method.

| **Electrode** | **Ø_B_ [meV]** |
| --- | --- |
| WO_3_/Cu/ ITO | ~ 220 |
| Cu/ITO | ~ 100 |
| Ni/ITO | ~ 60 |
| Ag/ITO | ~ 20 |
| Au/ITO | ~ 10 |
| ITO | ~ 5 |


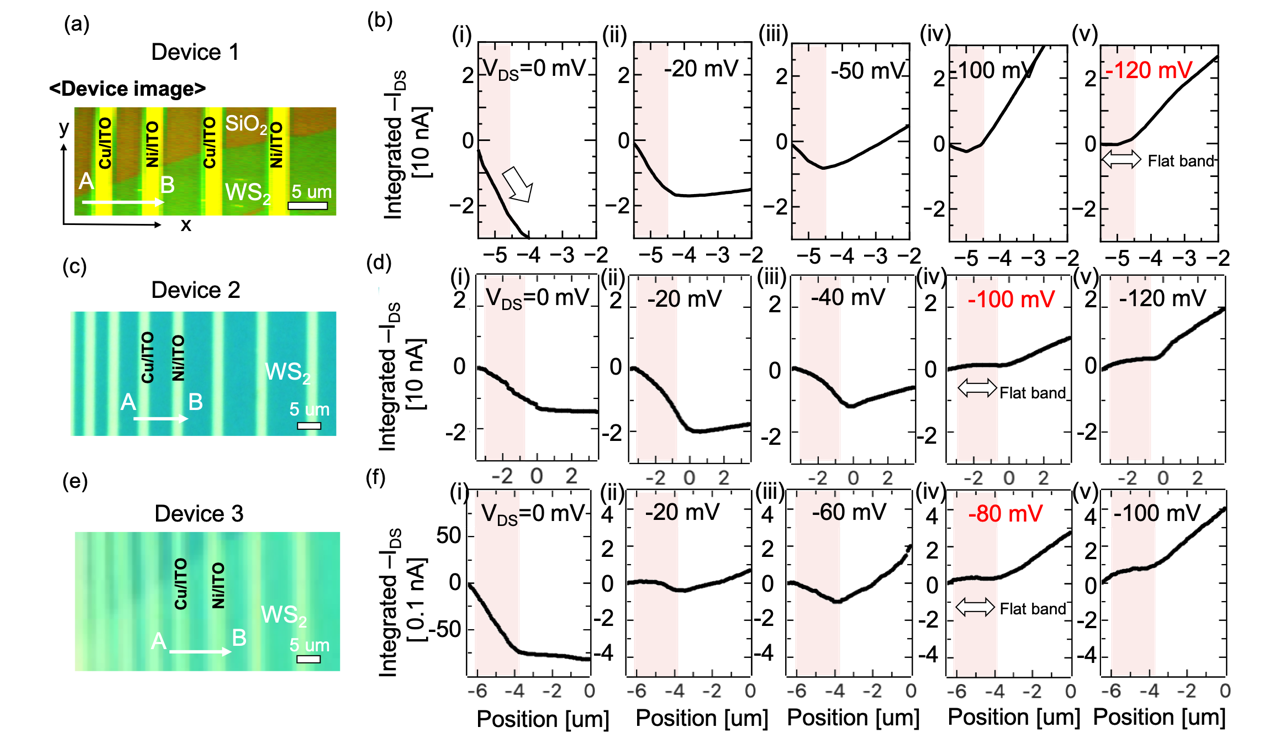


**Figure S1|** (a, c, e) Images of three devices with Cu/ITO and Ni/ITO as drain and source side, respectively. WS_2_ is channel material suspended on the electrodes. (b, d, f) (i–v) Profile of integrated I_DS_ at Cu/ITO side under different values of V_DS_ of the three devices.

During the Potential profile measurement, the line scan was performed multiple times, not only the x-axis dependence, we also changed the position of y-axis (Fig. S1 (a)), since the results showed almost the same, we only took a set of data. Furthermore, this experiment was performed with different devices, as shown in Fig. S1 (b), (d), (e), they all showed similar trends, i.e. near the Cu/ITO electrode, when V_DS_ = 0 meV, integrated I_DS_ shows a similar downward trend, which comes from the potential change of the Schottky barrier. After V_DS_ gradually increases, the rate of decline gradually decreases. When the V_DS_ matches the build-in potential (Schottky barrier), a flat band appears, which can be assumed to be Φ_B_, as shown in Fig. S1(a) and (b). For device 1, the flat band is shown at V_DS_ = -120 mV, and similarly, the flat band is shown at V_DS_ = -100 mV for device 2 (Fig. S1 (c) and (d)), and V_DS_ = -80 mV in device 3 (Fig. S1 (e) and (f), which means that the Φ_B_ of Cu/ITO in contact with WS_2_ is 80~120 meV.


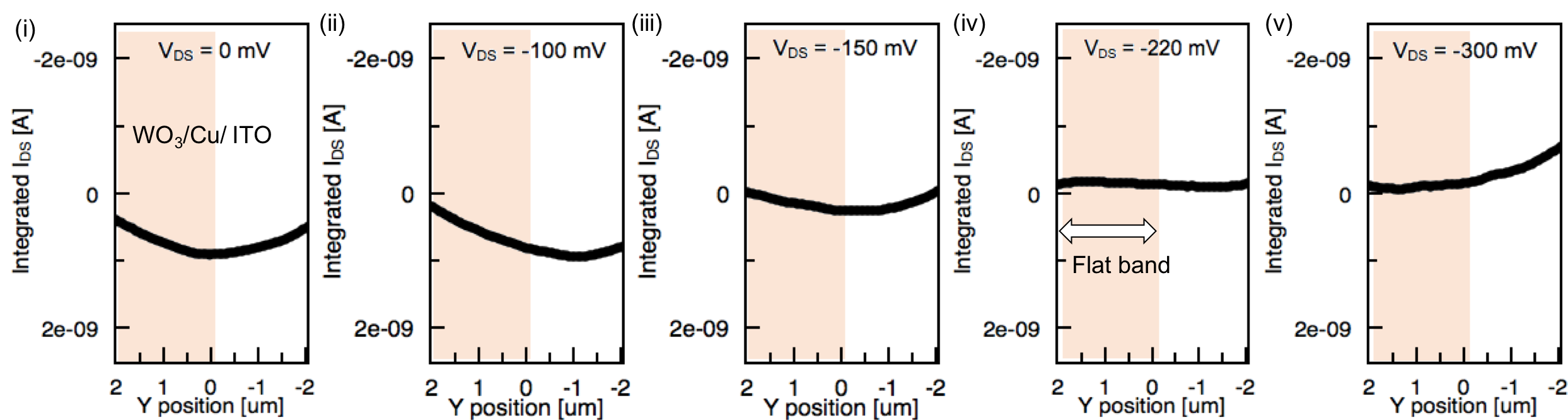


**Figure S2|** (i–v) Potential profile of WO_3_/Cu/ITO contact with WS_2_ with an inverse bias applied, V_DS_, and flat band forms when V_DS_ = –220 mV shown in (iv), indicating *Φ*_B_ is 220 meV between the contact of WO_3_/Cu/ITO and WS_2_.

**Figure captions:**

**Figure S1|** (i–v) Potential profile of WO_3_/Cu/ITO contact with WS_2_ with an inverse bias applied, V_DS_, and flat band forms when V_DS_ = –220 mV shown in (iv), indicating *Φ*_B_ is 220 meV between the contact of WO_3_/Cu/ITO and WS_2_.

**Figure S2| Performance of solar cells in um^2^ scale.** (a–d) Typical I-V curves of the solar cell with electrodes of ITO–ITO, WO_3_/ITO–ITO, Cu/ITO–ITO, and WO_3_/Cu/ITO–ITO, respectively. Scale bars in inset images represent 50 um. (e–g) Comparison of open circuit voltage, short circuit current, and fill factor, respectively, of the solar cells shown in (a–d).

**Figure S3| Devices with a simple enlarged pattern.** (a) Images of the devices, (b) device area calculation method, (c) normalized P_T_, and (d) open circuit voltage (V_OC_) of the devices with increasing area.

**Figure S4| Parallel connection equivalent circuit for the unit device.** (a) Unit device treated as many small channels (i, ii, iii, …) connected in parallel. (b) Total resistance (R_sh_) calculated from the equation 1/R_sh_ =$\Sigma$(1/R_sh_ (i)). (c) Schematic diagram of the device with a low parallel resistance part, which may come from impurities or metallic-like 1T phase.

**Figure S5| Dependency on the number of parallel connections (N_dev_) of unit device module A (UDM-A).** (a) Images of the device structure. (b, c) Images of the devices with same channel length and width, but different N_dev_. (d, e) Plots of normalized P_T_ and V_oc_ versus N_dev_; black line in (d) is the ideal tendency of normalized P_T_ and black dash line in (e) is the fitting curve of V_OC_.

**Figure S6| Design of UDM-B: seriers connection of UDM-A.** (a, b) Diagram of N_se_ designed for the device. (c, d, e) P_T_, V_OC_, I_SC_ of the devices with N_se_ values.

**Figure S7| Comparision of designed patteren and simple pattern.** (a) P_T_ of designed and simple patterns. (b–e) Typical I–V curve of the solar cells as shown in (a) (1)–(4). (f, g) Voc and I_SC_ of designed pattern and simple pattern.­

**Figure S8|** Images of (a) quartz, (b) electrodes on quartz, and (c) solar cell on quartz combined with electrodes and WS_2_.

**Figure S9| Photoluminencent intensity of CVD grown WS_2_.** (a) Schematic illustration of PL intensity testing and mapping. (b) PL mapping of WS_2_ sheet on SiO_2_/Si substrate, green dots represent monolayer WS_2_. (c) Image and (d) PL mapping of single crystal monolayer WS_2_ at the red cross in (b). (e) PL intensity of WS_2_ in (d).


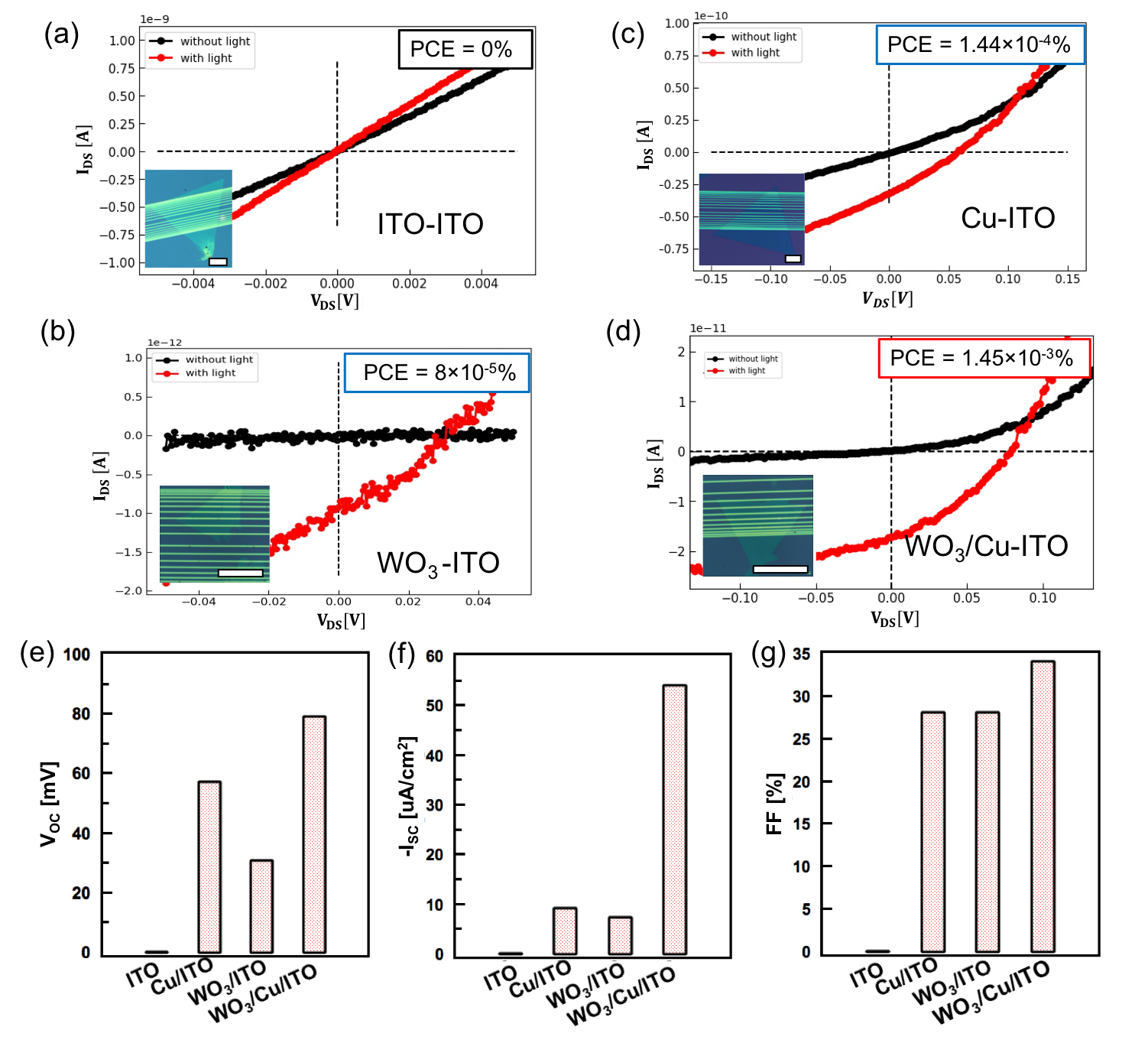


**Figure S3| Performance of solar cells in um^2^ scale.** (a–d) Typical I-V curves of the solar cell with electrodes of ITO–ITO, WO_3_/ITO–ITO, Cu/ITO–ITO, and WO_3_/Cu/ITO–ITO, respectively. Scale bars in inset images represent 50 um. (e–g) Comparison of open circuit voltage, short circuit current, and fill factor, respectively, of the solar cells shown in (a–d).


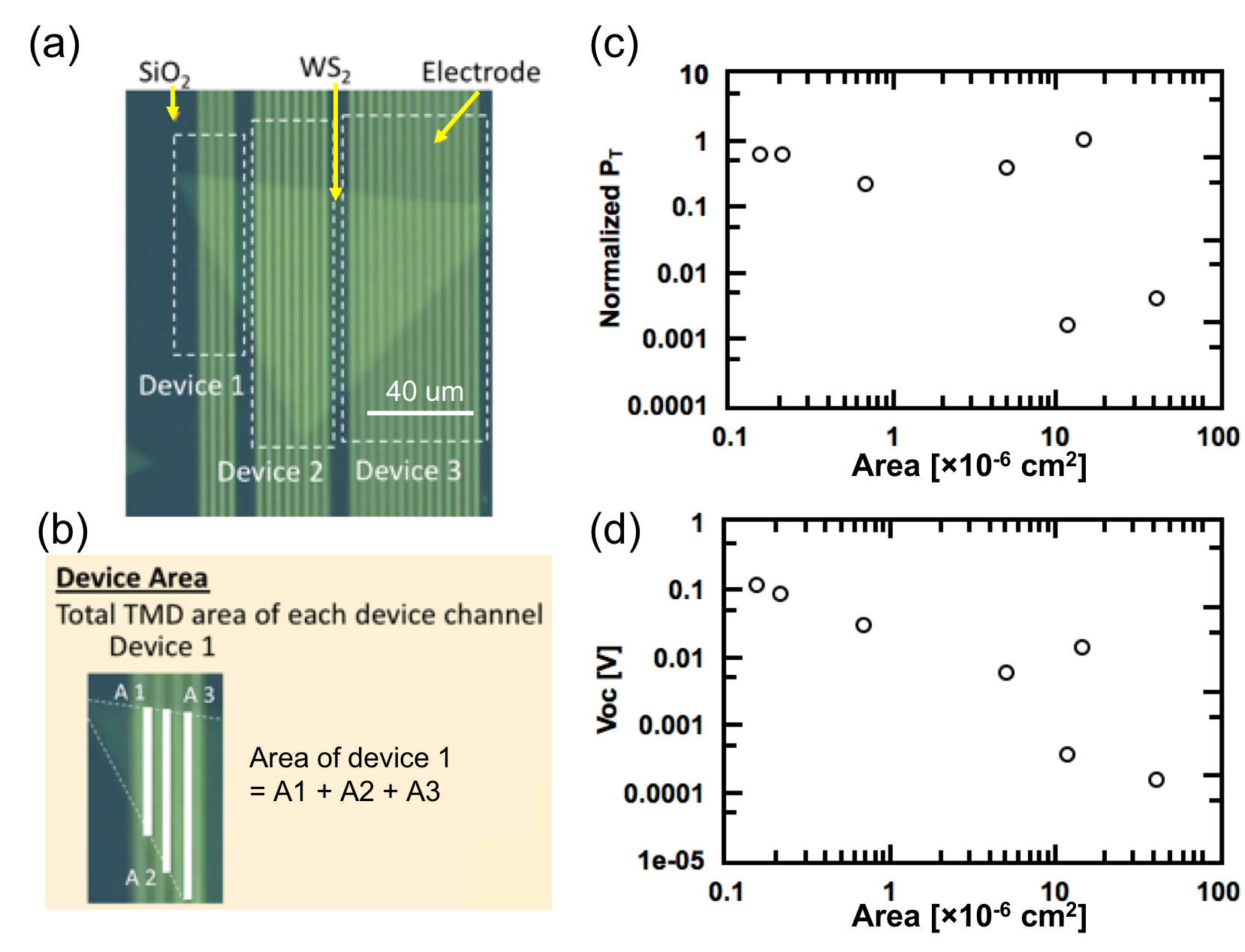


**Figure S4| Devices with a simple enlarged pattern.** (a) Images of the devices, (b) device area calculation method, (c) normalized P_T_, and (d) V_OC_ of the devices with increasing area.


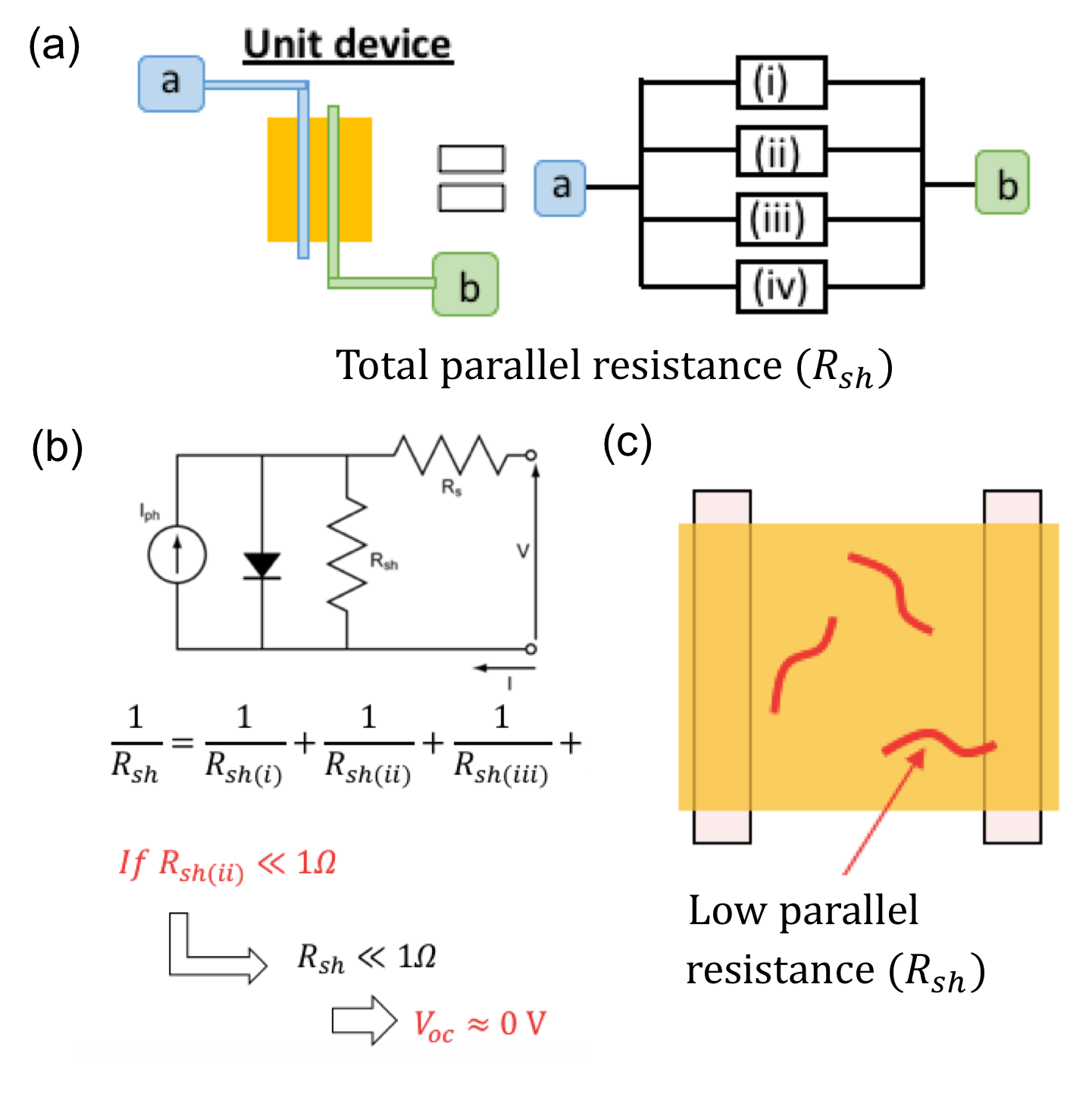


**Figure S5| Parallel connection equivalent circuit for the unit device.** (a) Unit device treated as many small channels (i, ii, iii, …) connected in parallel. (b) Total parallel (shunt) resistance (R_sh_) calculated from the equation 1/R_sh_ =$\Sigma$(1/R_sh_ (i)). (c) Schematic diagram of the device with a low parallel resistance part, which may come from impurities or metallic-like 1T phase.


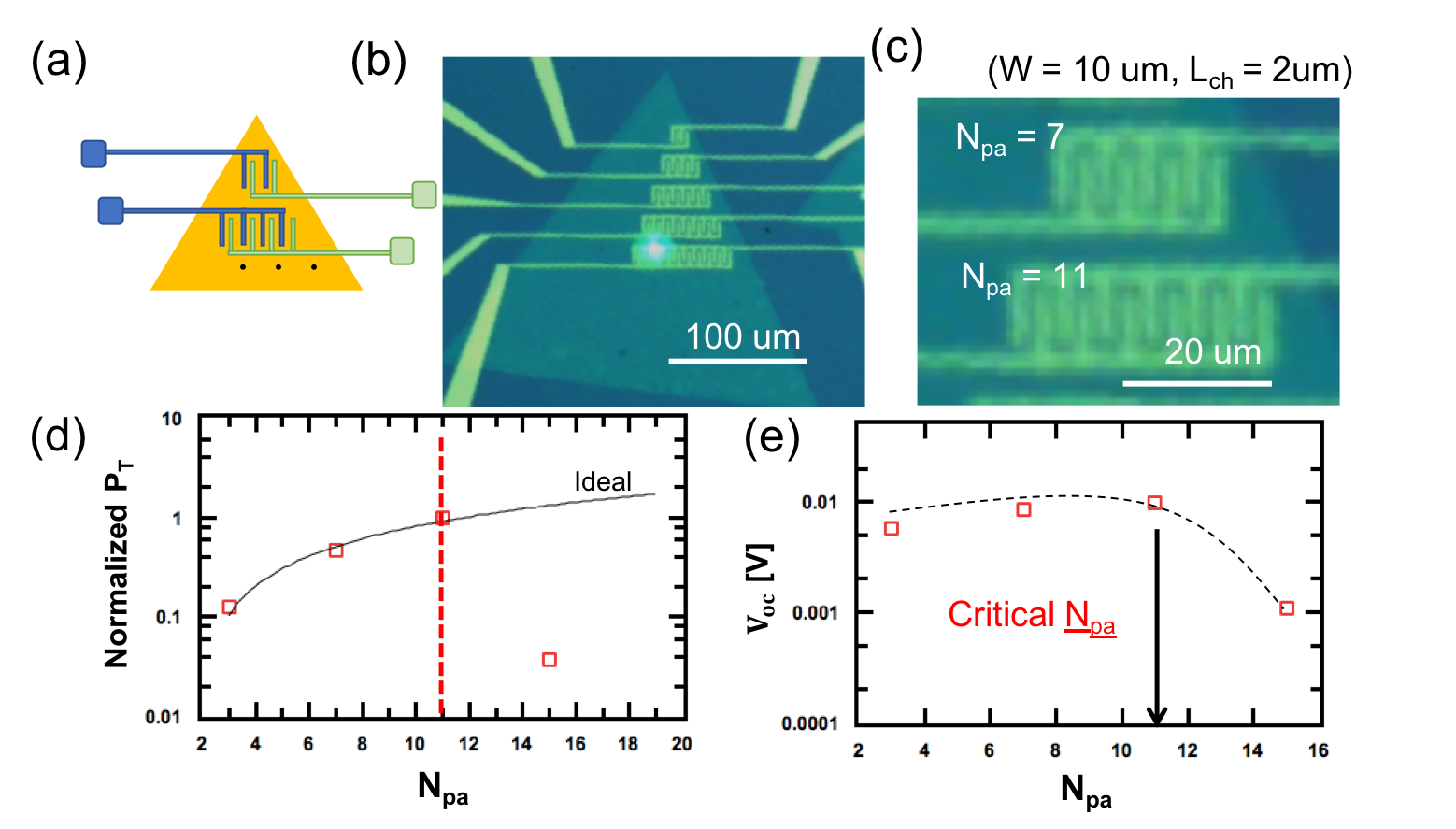


**Figure S6| Dependency on the number of parallel connections (N_pa_) of unit device module A (UDM-A).** (a) Images of the device structure. (b, c) Images of the devices with same channel length and width, but different N_pa_. (d, e) Plots of normalized P_T_ and V_OC_ versus N_pa_; black line in (d) is the ideal tendency of normalized P_T_ and black dash line in (e) is the fitting curve of V_OC_.


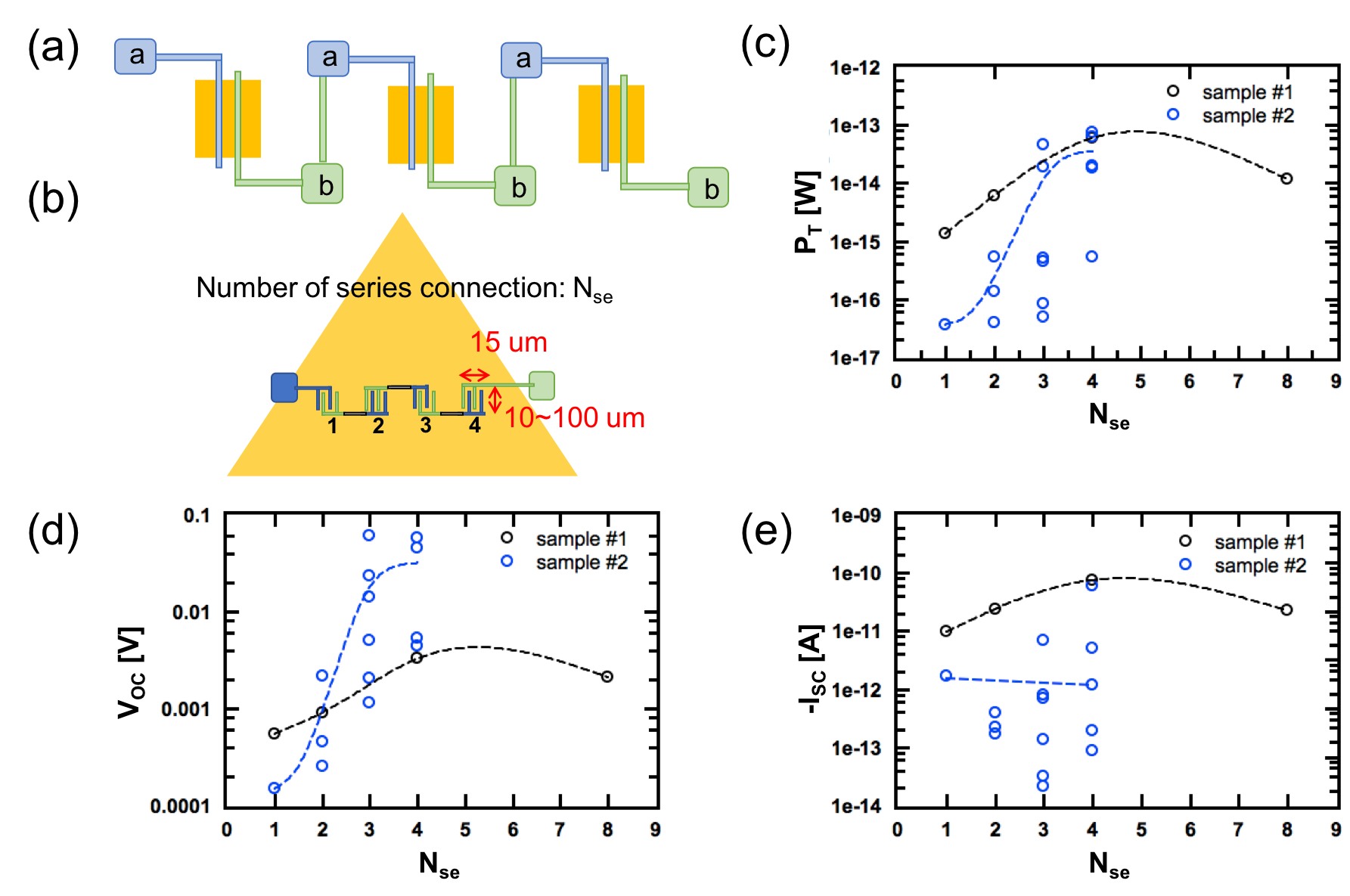


**Figure S7| Design of UDM-B: seriers connection of UDM-A.** (a, b) Diagram of N_se_ designed for the device. (c, d, e) P_T_, V_OC_, I_SC_ of the devices with N_se_ values.


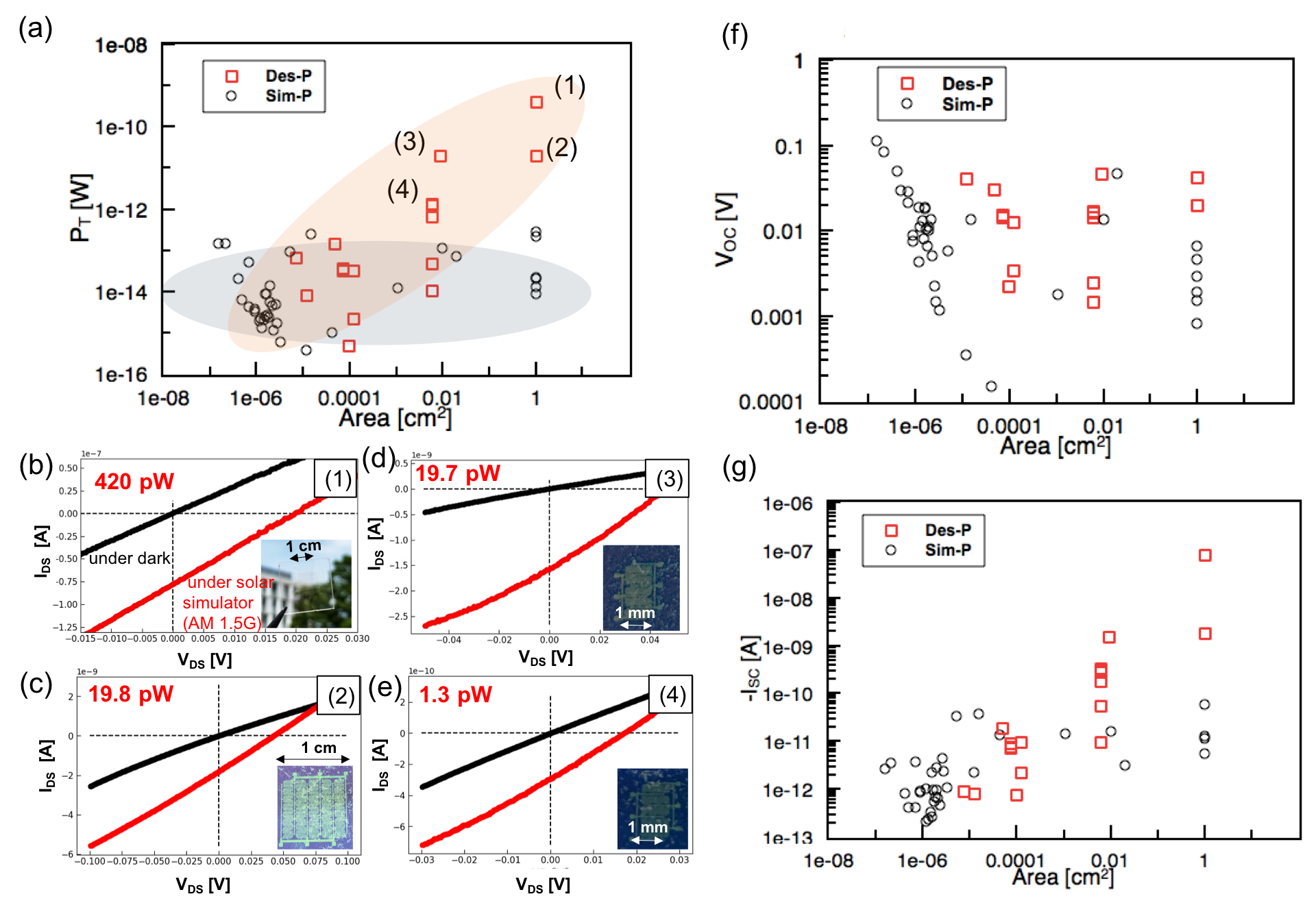


**Figure S8| Comparision of designed patteren and simple pattern.** (a) P_T_ of designed and simple patterns. (b–e) Typical I–V curve of the solar cells as shown in (a) (1)–(4). (f, g) V_OC_ and I_SC_ of designed pattern and simple pattern.


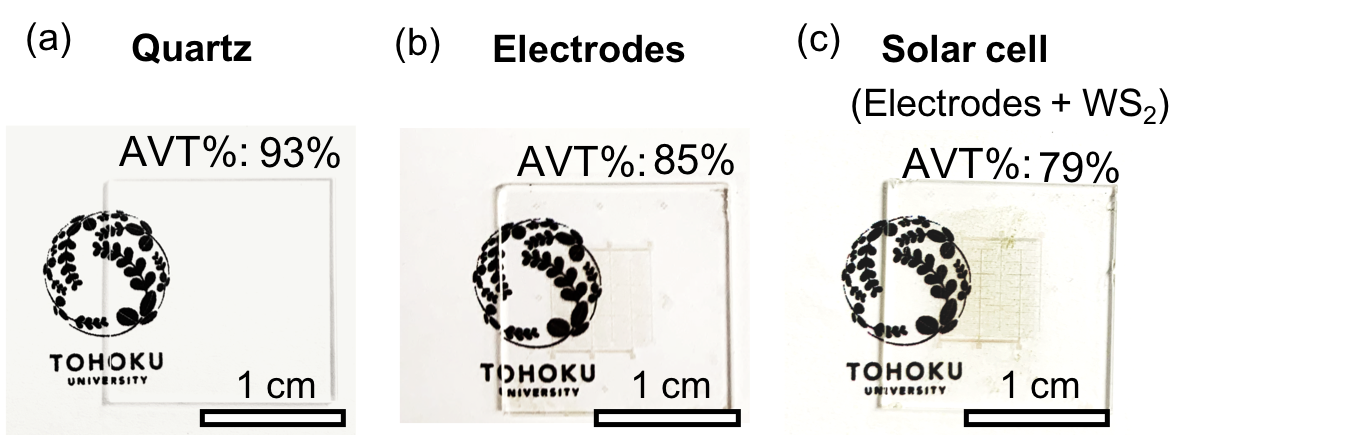


**Figure S9|** Images of (a) quartz, (b) electrodes on quartz, and (c) solar cell on quartz combined with electrodes and WS_2_.


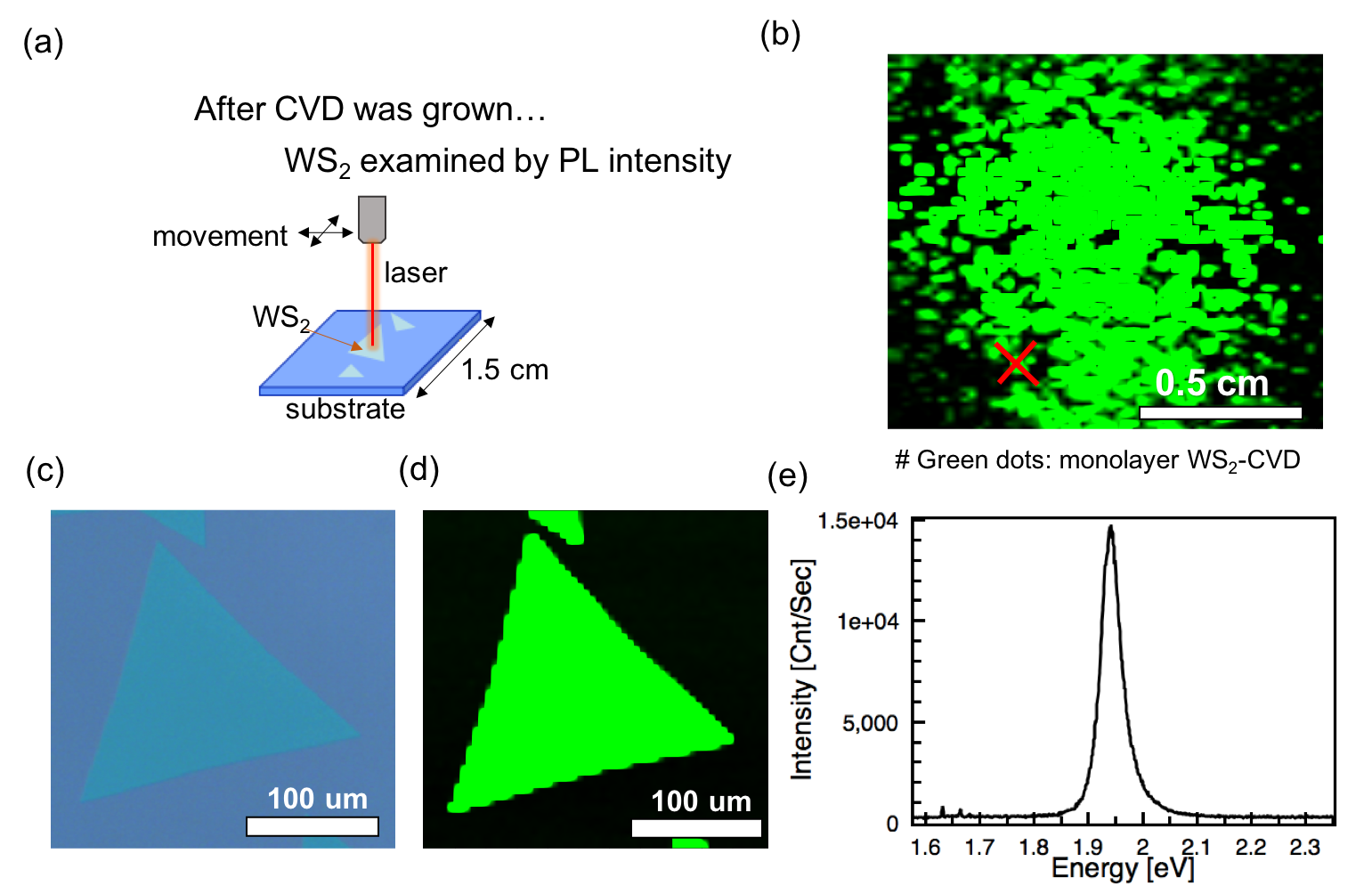


**Figure S10| Photoluminencent intensity of CVD grown WS_2_.** (a) Schematic illustration of PL intensity testing and mapping. (b) PL mapping of WS_2_ sheet on SiO_2_/Si substrate, green dots represent monolayer WS_2_. (c) Image and (d) PL mapping of single crystal monolayer WS_2_ at the red cross in (b). (e) PL spectrum of WS_2_ in (d).

The defect and impurities of WS_2_ can have an impact on the performance of solar cells. For example, defects inside the band gap with deep transition can entice carriers, which results into Shockley–Read–Hall non-radiative recombination centers. Metallic 1T phase or impurities that cause metallic pass could reduce V_OC_. In our group, at current state, we can grow large area monolayer WS_2_ on a 1x1 cm^2^ Si/SiO_2_ or quartz substrate, the WS_2_ is single crystal and obvious pinholes can not identified in our high quality sample.


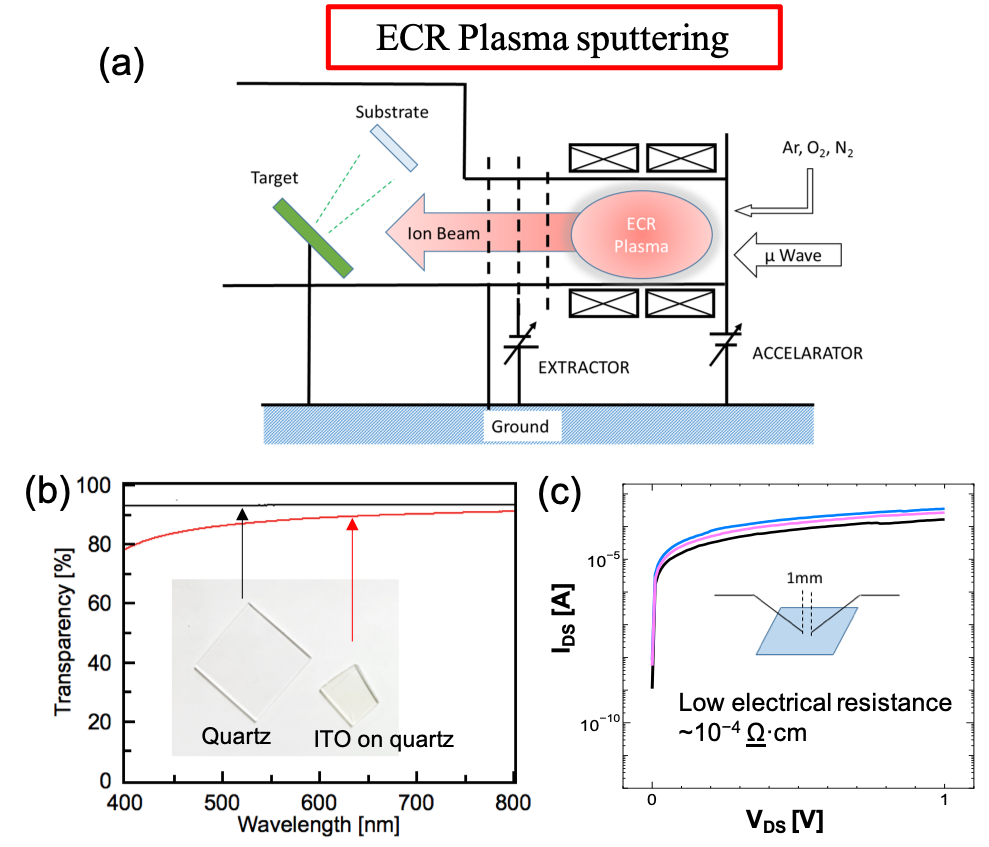


**Figure S11|** (a) Scheme of ECR plasma sputtering, (b) transparency of with/without ITO on quartz substrate, (c) typical I-V curve for calculating electrical resistance of ITO.

The ITO used in this work was explored under different sputtering conditions, and finally the condition with the highest electrical conductivity was selected, which also means that the quality and surface morphology of the electrode material under this condition are the most favorable for the fabrication of solar cells (Figure S11).

The morphology and quality of the ITO or thin layer metal/metal oxides have an impact on the performance of solar cells. For example, if the surface morphology is uneven and rough, it will reduce the contact area, increase the series resistance, and reduce the amount of carriers, but the effect on V_OC_ will not be significant. Regarding pinholes, I think that in the structure of vertical solar cells, the presence of a pinhole defects through these layers would lead to the formation of a wire-like connection through the photovoltaic device (shunt), which would seriously degrade the overall performance of the photovoltaic device, on deposition of the metal back contact. However, in our planar structure, the influence of pinholes may be not so significant.

**References**

S1. Memaran, S. *et al.* Pronounced photovoltaic response from multilayered transition-metal dichalcogenides pn-junctions. *Nano Lett.* **15**, 7532–7538 (2015).

S2. Wi, S. *et al.* Enhancement of photovoltaic response in multilayer MoS_2_ induced by plasma doping. *ACS Nano* **8**, 5270–5281 (2014).

S3. Cho, A. J., Song, M. K., Kang, D. W. & Kwon, J. Y. Two-dimensional WSe_2_/MoS_2_ p-n heterojunction-based transparent photovoltaic cell and its performance enhancement by fluoropolymer passivation. *ACS Appl. Mater. Interfaces* **10**, 35972–35977 (2018).

S4. Pawar, S. A. *et al.* Heterojunction solar cell based on n-MoS_2_/p-InP. *Opt. Mater.* **86**, 576–581 (2018).

S5. Nazif, N. K. *et al.* High-performance p-n junction transition metal dichalcogenide photovoltaic cells enabled by MoO_x_ doping and passivation. *Nano Lett.* **21**, 3443–3450 (2021).

S6. Fontana, M. *et al.* Electron-hole transport and photovoltaic effect in gated MoS_2_ Schottky junctions. *Sci. Rep.* **3**, 1634–8–13 (2013).

S7. McVay, E., Zubair, A., Lin, Y., Nourbakhsh, A. & Palacios, T. Impact of Al_2_O_3_ passivation on the photovoltaic performance of vertical WSe_2_ Schottky junction solar cells. *ACS Appl. Mater. Interfaces* **12**, 57987–57995 (2020).

S8. Groenendijk, D. J. *et al.* Photovoltaic and photothermoelectric effect in a double-gated WSe_2_ device. *Nano Lett.* **14**, 5846–5852 (2014).

S9. Pospischil, A., Furchi, M. M. & Mueller, T. Solar-energy conversion and light emission in an atomic monolayer p-n diode. *Nat. Nanotechnol.* **9**, 257–261 (2014).

S10. Wang, L. & Sambur, J. B. Efficient ultrathin liquid junction photovoltaics based on transition metal dichalcogenides. *Nano Lett.* **19**, 2960–2967 (2019).

S11. Akama, T. *et al.* Schottky solar cell using few-layered transition metal dichalcogenides toward large-scale fabrication of semitransparent and flexible power generator. *Sci. Rep.* **7**, 11967–1–10 (2017).
